# *Croton campestris* A. St.-Hill Methanolic Fraction in a Chlorpyrifos-Induced Toxicity Model in *Drosophila melanogaster*: Protective Role of Gallic Acid

**DOI:** 10.1155/2020/3960170

**Published:** 2020-03-22

**Authors:** Karen Kich Gomes, Giulianna Echeverria Macedo, Nathane Rosa Rodrigues, Cynthia Camila Ziech, Illana Kemmerich Martins, Jéssica Ferreira Rodrigues, Patrícia de Brum Vieira, Aline Augusti Boligon, Francisco Elizaudo de Brito Junior, Irwin R. A. de Menezes, Jeferson Luis Franco, Thaís Posser

**Affiliations:** ^1^Oxidative Stress and Cell Signaling Research Group, Interdisciplinary Research Center on Biotechnology-CIPBIOTEC, Universidade Federal do Pampa, Campus São Gabriel, RS, Brazil; ^2^Department of Chemistry, Post Graduate Program in Toxicological Biochemistry, Universidade Federal de Santa Maria, RS, Brazil; ^3^Postgraduate Program in Pharmaceutical Sciences, Universidade Federal de Santa Maria, Santa Maria, Rio Grande do Sul, Brazil; ^4^Department of Biological Chemistry, Universidade Regional do Cariri, Crato, CE, Brazil

## Abstract

*Croton campestris* A. St-Hill popularly known as “velame do campo” is a native species of the savannah from northeastern Brazil, being used in folk medicine due to its beneficial effects in the treatment of many diseases, inflammation, detoxification, gastritis, and syphilis; however, its potential use as an antidote against organophosphorus compound poisoning has not yet been shown. Here, the protective effect of the methanolic fraction of *C. campestris* A. St.-Hill (MFCC) in *Drosophila melanogaster* exposed to chlorpyrifos (CP) was investigated. Flies were exposed to CP and MFCC during 48 h through the diet. Following the treatments, parameters such as mortality, locomotor behavior, and oxidative stress markers were evaluated. Exposure of flies to CP induced significant impairments in survival and locomotor performance. In parallel, increased reactive oxygen species and lipoperoxidation occurred. In addition, the activity of acetylcholinesterase was inhibited by CP, and superoxide dismutase and glutathione S-transferase activity was induced. Treatment with MFCC resulted in a blockage of all CP-induced effects, with the exception of glutathione S-transferase. Among the major compounds found in MFCC, only gallic acid (GA) showed a protective role against CP while quercetin and caffeic acid alone were ineffective. When in combination, these compounds avoided the toxicity of CP at the same level as GA. As far as we know, this is the first study reporting the protective effect of MFCC against organophosphate toxicity *in vivo* and highlights the biotechnological potential of this fraction attributing a major role in mediating the observed effects to GA. Therefore, MFCC may be considered a promising source for the development of new therapeutic agents for the treatment of organophosphate intoxications.

## 1. Introduction

Agriculture has undergone a process of expansion and increase in productivity in the last decades in order to attend to the growing demand for agricultural products. The expansion of agriculture is followed by the adoption of modern technologies and also the massive use of agrochemicals which has become a worldwide environmental and public health concern. The occupational exposure and consumption of agrochemical residues have been associated with a larger incidence of neurodegenerative diseases [1].

Chlorpyrifos (0,0-Diethyl-O-[3,5,6-trichloro-2-pyridyl]phosphorothioate) (CP) belongs to the organophosphorus (OP) class and is an insecticidal anticholinergic agent used for agriculture and domestic purpose [2, 3]. The CP acts by inhibiting the enzyme acetylcholinesterase (AChE), resulting in the accumulation of acetylcholine in the synaptic cleft, causing hyperstimulation of nicotinic and muscarinic receptors, and disruption of neurotransmission [4, 5].

In parallel to AChE inhibition, CP exposure modulates cellular redox status leading to an oxidative stress condition, characterized by an imbalance between reactive oxygen species (ROS) levels and cellular antioxidant defense able to contrapose those species [6]. ROS are generated in a natural way as a part of the cell metabolism, regulating an array of signal transduction pathways when present in transient quantities; in excess, they can lead to cellular damage to DNA, protein, and lipids [7]. The causes leading to oxidative stress by CP involve modulation of antioxidant enzymes and inhibition of mitochondrial complex I [8–10].

Treatment for OP intoxication includes the use of atropine (anticholinergic), oximes (AChE reactivators), and benzodiazepines (anticonvulsants) [11]. However, these treatments are often inefficient [12]. Thus, studies focused on the search for alternative treatments for PO poisoning are of relevance.

Brazil has great vegetal biodiversity, comprising 15 to 25% of the world's total plant species [13]. The species *C. campestris* A. St.-Hill belongs to the family Euphorbiaceae, being a native bush species of the Cerrado biome in northeastern Brazil. The genus *Croton* is composed of 700 species and is distributed in tropical regions. It is popularly known as “velame do campo” and is used through infusions of leaves and roots of the plant in alcoholic beverages and teas and is used in Brazilian folk medicine for gastric, hematological, and inflammatory disorders, as well as respiratory problems [14–16]. Substantial evidence from the literature indicates that *Croton campestris* A. St.-Hill displays antibacterial [14, 15], antibiotic [15, 17], molluscicidal [18], and anti-inflammatory properties [19]. Recently, our research group reported the antiulcerogenic activity of the leaf extracts of *Croton campestris* A. St.-Hill [16]. However, the possible mechanism by which *Croton campestris* A. St.-Hill exerts this action was not clearly understood. In a previous study, our group demonstrated that the crude extract of *C. campestris* A. St.-Hill presented toxicity and prooxidant effects on *Drosophila melanogaster* [20]. However, the effects of the methanolic fraction of *C. campestris* A. St.-Hill (MFCC) are unknown.

Phenolic compounds comprise a large group of phytochemicals, such as phenolic acids, flavonoids, and tannins which are present ubiquitously in plants as secondary metabolites. A wide range of biological properties including those of biomedical interest such as antioxidant, anti-inflammatory, and antimicrobial activity have been attributed to these compounds [21]. Moreover, protective potential of flavonoids against CP-induced damage has been already described [22, 23]. Therefore, the potential protective effect of MFCC against OP-induced toxicity remains to be elucidated.

In the present study, the protective potential of the methanolic fraction of *C. campestris* A. St.-Hill against toxicity induced by the organophosphate chlorpyrifos in the fruit fly *Drosophila melanogaster* was evaluated.Additionally, the potential of major phenolic compounds detected in the fraction by HPLC was evaluated in an isolated or collective manner against the toxicity induced by CP.

## 2. Material and Methods

### 2.1. Chemicals

All the chemicals used in the study were purchased from Sigma-Aldrich (São Paulo, SP, Brazil).

### 2.2. Plant Collection and Extraction

The leaves of *C. campestris* A. St.-Hill were collected in the municipality of Crato, Ceará, Brazil (7°22′2.8^″^S, 39°28′42.4^″^W, altitude: 892 m above sea level) in the year 2011. Its identification was carried out at the Federal University of Rio Grande do Norte (UFRN). Dried leaves (2200 g) were macerated in 6.5 L of 99.9% of ethanol and water (1 : 1, *V*/*V*) and allowed to rest for 7 days. After filtration, the solvent was evaporated and the remained extract was lyophilized to obtain 12.6 g of the hydroalcoholic extract of the leaves of *C. campestris* A. St.-Hill. From this extract, the methanolic fraction of *C. campestris* A. St.-Hill (MFCC) was prepared as described elsewhere [24].

### 2.3. Quantification of Compounds by High-Performance Liquid Chromatography with Diode Array Detection (HPLC-DAD)

Reverse-phase chromatographic analysis was carried out under gradient conditions using a C18 column (4.6 mm × 250 mm, 5 *μ*m) as described by Boligon et al. [25]. Firstly, the methanolic fraction of *C. campestris* A. St.-Hill was dissolved in ethanol at a concentration of 2 mg/mL. The presence of eight compounds was detected: gallic acid, chlorogenic acid, caffeic acid, catechin, quercetin, quercitrin, rutin, and kaempferol. Identification of these compounds was performed by comparing their retention time and UV absorption spectrum with those of the commercial standards. The flow rate was 0.7 mL/min, and stock solutions of standard references were prepared in the HPLC mobile phase at a concentration range of 0.030–0.250 mg/mL for kaempferol, quercetin, quercitrin, catechin, and rutin and 0.030–0.250 mg/mL for gallic, caffeic, and chlorogenic acids. All chromatography operations were carried out at ambient temperature and in triplicate. The limit of detection (LOD) and limit of quantification (LOQ) were calculated based on the standard deviation of the responses and the slope using three independent analytical curves, as described elsewhere [26].

### 2.4. *Drosophila* Stock and Culture

For this study, female flies (*Drosophila melanogaster*, Harwich lineage) were used. The flies were reared under controlled humidity (60-70%) and temperature (25°C) in a 12-hour light/dark cycle and kept in glass tubes measuring 50 mm × 85 mm and containing 10 mL of standard medium (corn flour, salt, wheat germ, powdered milk, sugar, soy flour, and rye flour) supplemented with dry yeast. Nipagin® was used as an antifungal agent as described elsewhere [27].

### 2.5. Experimental Design

For the experiments, female flies (1–4 days old) were divided into 4 groups of 40 flies in triplicate totaling 160 flies *per* treatment and exposed to solutions in soaked cotton wool. The experimental groups were as follows: control group (1% sucrose), MFCC group (treated with 0.1 mg/mL methanolic fraction dissolved in 1% sucrose), CP group (treated with 0.25 ppm CP diluted in 1% sucrose), and CP/MFCC cotreatment group. CP stock solutions were prepared in absolute ethanol, and the final ethanol concentration in all groups containing CP was 0.09% and did not show any changes in the tested parameters (unpublished results). All treatments lasted 48 hours. Survivorship was recorded after 24 and 48 hours. Survived flies were then subjected to behavioral and biochemical assays. The CP concentrations used in this study were based on a survival curve (Supplementary [Supplementary-material supplementary-material-1]).

### 2.6. Locomotor Activity

The locomotor activity was assayed as the negative geotaxis test (climbing capacity) described by Bland et al. [28] with some modifications. Briefly, after treatments were finished, 10 flies *per* group were anesthetized in ice and placed in vertical glass tubes (length 25 cm, diameter 1.5 cm) closed with cotton. After 30 min of recovery, the flies were pulled to the bottom of the tube and then the number of flies able to climb 5 cm in the flask was registered after 6 seconds. The test was repeated 3 times in intervals of 20 s each. Thirty flies were used *per* group (*n* = 30). All experiments were carried out in triplicate. The results were expressed as the percentage of control.

### 2.7. Enzyme Assays

To determine the enzyme activities, twenty flies *per* group were used. The whole flies were homogenized in 20 mM HEPES (pH 7.0) and centrifuged at 1000 × g for 5 minutes at 4°C. An aliquot of supernatant was separated for acetylcholinesterase (AChE) activity according to Ellman et al. as described elsewhere [29]. The remaining supernatant was centrifuged at 20000 × g for 30 min at 4°C for measurements of superoxide dismutase (SOD) [30], catalase (CAT) [31], and glutathione S-transferase (GST) [32]. Protein concentration was determined by the method of Bradford [33]. 200 flies were used *per* group, which were divided into 4–9 biological replicates, performed in triplicate. Enzyme activity was expressed as mU/mg/protein.

### 2.8. Determination of Cellular Viability and Arbitrary Steady-State ROS Levels

When treatments were finished, a number of twenty flies *per* group were homogenized in 1 mL of mitochondrial isolation buffer (220 mM mannitol, 68 mM sucrose, 10 mM KCl, 10 mM HEPES, and 1% BSA) following centrifugation at 1000 × g for 10 min (4°C). The mitochondria-enriched supernatant was used for determination of cellular viability through mitochondrial activity (resazurin assay; 544 nm_ex_/590 nm_em_) analysis, and the redox steady state was measured with the fluorescent dye 2,7-dichlorofluorescein diacetate (DCF-DA; 485 nm_ex_/530 nm_em_). The assays were performed according to previously described protocols [34]. All assays were conducted in triplicate. The data were standardized by protein concentration and expressed as the percentage of the control group.

### 2.9. Lipid Peroxidation

The lipid peroxidation was measured by quantification of malondialdehyde (MDA) by HPLC as described by Saraiva et al. [35]. The retention time of MDA was 4.375-4.709 min. The preparation of MDA standards was performed as described by Karatas et al. [36], and the MDA was detected by comparison with the standard curve and the results were expressed as *μ*g/mL (mean ± SEM).

### 2.10. Protective Effects of Active Compounds of the Methanolic Fraction of *Croton campestris* A. St.-Hill

Among the compounds found by HPLC analysis, gallic acid, caffeic acid, and quercetin were assayed in an isolated and collective manner, to test the possible protection against the toxicity of CP. The concentrations used for each compound were based on their quantities revealed in the chromatographic profile of the MFCC. The concentration of gallic acid, caffeic acid, and quercetin was 1.2 mg/mL, 2.5 mg/mL, and 3 mg/mL, respectively.

### 2.11. IBR Calculation

Parameters of toxicity described in Material and Methods for different treatments were combined into a stress index termed “integrated biomarker response” (IBR) described by Beliaeff and Burgeot [37].

### 2.12. Statistical Analysis

Data were subjected to D'Agostino and Pearson, Shapiro-Wilk, and Kolmogorov-Smirnov normality tests. The parametric data were expressed as mean ± standard error mean (SEM) and were analyzed by one-way ANOVA followed by the Newman-Keuls *post hoc* test, and nonparametric data were expressed as median (interquartile range) and analyzed by the Kruskal-Wallis test followed by Dunn's *post hoc* test. The results were considered statistically significant when *p* ≤ 0.05. LC_50_ was calculated using Probit analysis.

## 3. Results

### 3.1. Identification and Quantification of Phenolic Compounds of the *Croton campestris* A. St.-Hill Methanolic Fraction by High-Performance Liquid Chromatography with Diode Array Detection (HPLC-DAD)

**Figure 1:**
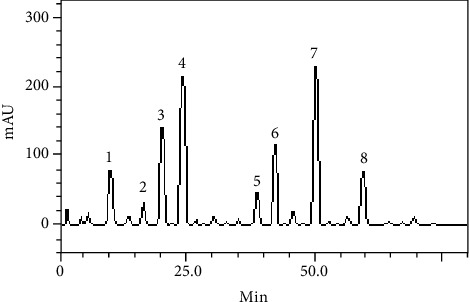
Representative high-performance liquid chromatography profile of *Croton campestris*. Detection UV was at 325 nm. Gallic acid (peak 1), catechin (peak 2), chlorogenic acid (peak 3), caffeic acid (peak 4), rutin (peak 5), quercitrin (peak 6), quercetin (peak 7), and kaempferol (peak 8). Chromatographic conditions are described in Material and Methods.

HPLC fingerprinting of the *C. campestris* A. St.-Hill methanolic fraction revealed the presence of gallic acid (tR = 10.13 min, peak 1), catechin (tR = 16.78 min, peak 2), chlorogenic acid (tR = 20.15 min, peak 3), caffeic acid (tR = 24.63 min, peak 4), rutin (tR = 38.17 min, peak 5), quercitrin (tR = 42.59 min, peak 6), quercetin (tR = 50.11 min, peak 7), and kaempferol (tR = 59.84 min, peak 8) (Figure 1 and Table 1). The HPLC analysis revealed that flavonoids (quercetin, quercitrin, rutin, and kaempferol), tannins (catechin), and phenolic acids (gallic, chlorogenic, and caffeic acids) are present. The major compounds were quercetin (30.29 mg/g), caffeic acid (27.64 mg/g), and chlorogenic acid (19.25 mg/g) (Figure 1) (Table 1).

### 3.2. Methanolic Fraction of *Croton campestris* A. St.-Hill Does Not Alter Mortality of Flies

The toxicity associated with the uptake of MFCC by adult flies was evaluated by a dose-response curve. As observed in supplementary [Supplementary-material supplementary-material-1], the fraction did not induce significant mortality of flies in the course of treatment (Supplementary [Supplementary-material supplementary-material-1]).

### 3.3. Dose-Response Curve of Chlorpyrifos

The concentration of CP used in this study was based on a concentration curve when the concentration able to cause about 50% of mortality (0.25 ppm) was chosen for further studies. LC_50_ found by Probit analysis was 0.21 ppm for 40 female flies after 48 h of treatment (Supplementary [Supplementary-material supplementary-material-1]).

### 3.4. Methanolic Fraction of *Croton campestris* A. St.-Hill Protects against Fly Mortality and Locomotor Deficits

**Figure 2:**
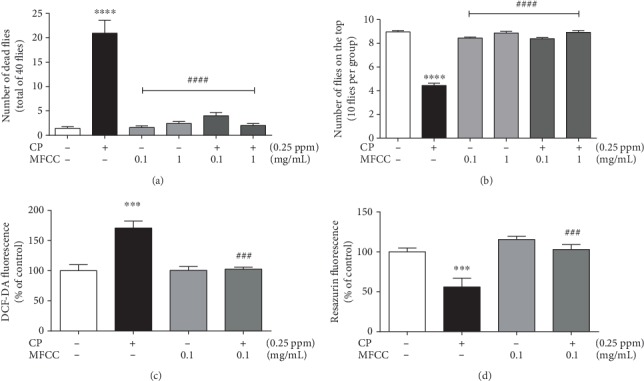
Effects of exposure to CP and MFCC on mortality (a), locomotor performance (b), ROS generation (c), and cell viability (d) in *Drosophila melanogaster*. The mortality score and negative geotaxis were evaluated after 48 hours of treatment in the presence of MFCC (0.1 and 1 mg/mL) and CP (0.25 ppm). ROS levels and cell viability were measured after 48 hours of treatment with MFCC (0.1 mg/mL) and CP (0.25 ppm). The results are represented as mean ± standard error of the mean (SEM), are expressed as mean ± SEM of the fluorescence levels of DCF-DA and resorufin, and are expressed as the percentage of control. The statistical analysis was performed by one-way ANOVA and the Newman-Keuls post hoc test. ^∗∗∗^*p* < 0.001 and ^∗∗∗∗^*p* < 0.0001 in relation to control and ^###^*p* < 0.001 and ^####^*p* < 0.0001 in relation to group CP.

We investigated the protective effect of MFCC on CP toxicity (0.25 ppm) in locomotor performance and fly mortality. CP-induced fly mortality was partially avoided by MFCC at 0.01 mg/mL, and total protection was observed from 0.1 mg/mL (Figure 2(a)). The exposure to CP induced important locomotor deficits in the flies; as can be visualized in the graph, only about 4 flies from a total of 10 per group were able to reach the top of the column. The addition of MFCC to the medium avoided totally the toxicity of CP on this parameter from 0.1 mg/mL (Figure 2(b)).

### 3.5. Reactive Oxygen Species (ROS) Generation and Cellular Viability

The potential of CP to induce ROS in the samples was evaluated in the presence or absence of a fraction of *C. campestris* A. St.-Hill. CP induced an increase in the fluorescence of the compound DCF-DA signaling for an augmented ROS generation in flies exposed to this compound. ROS generation was increased by 50% by CP and unchanged in relation to control when MFCC was present (Figure 2(c)). Similarly, the CP leads to a decay of 50% in mitochondrial viability, signaling for a drop in cell viability, represented by fluorescence of the compound resazurin (Figure 2(d)). This effect was reestablished in the presence of MFCC 0.1 mg/mL.

### 3.6. Malondialdehyde (MDA) Quantification by High-Performance Liquid Chromatography with Diode Array Detection (HPLC-DAD)

**Figure 3:**
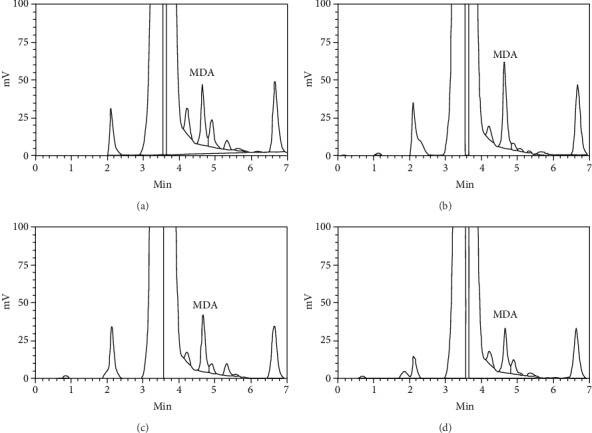
Representative chromatograms of malondialdehyde (MDA) analysis obtained in 30 mM KH_2_PO_4_-methanol (65 : 35, *v*/*v*%). The peak identification was performed by cochromatography with authentic standards. (a) Control. (b) CP-treated flies. (c) Flies treated with the methanolic fraction of *Croton campestris* (MFCC). (d) Cotreatment with CP and MFCC.

Peroxidation of polyunsaturated fatty acids (PUFAs) results in a variety of aldehydes, and MDA is the most abundant and best studied of these molecules. HPLC is a robust technique chosen because it is used to separate the authentic (TBA)_2_-MDA adduct from other chromogens that absorb at the same wavelength [38]. In the present study, the treatment with CP increased MDA levels by about 40% (Figure 3(b)). This increase in MDA levels in relation to control was not observed when MFCC was coincubated with CP, indicating a protective effect of the fraction (Figure 3(d)). The fraction per se did not alter MDA levels (Figure 3(c)).

### 3.7. Activity of Enzymes Acetylcholinesterase (AChE), Superoxide Dismutase (SOD), Glutathione S-Transferase (GST), and Catalase (CAT)

**Figure 4:**
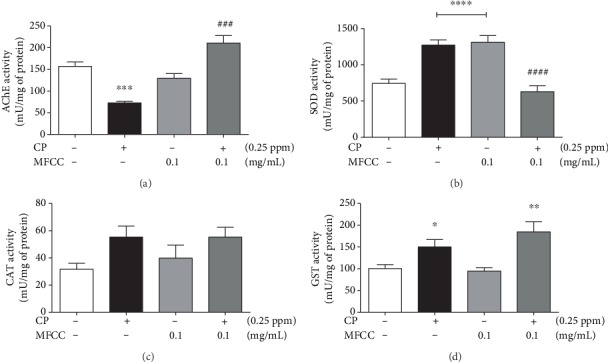
Activity of enzymes AChE, SOD, catalase, and GST in *Drosophila melanogaster* exposed to CP and MFCC. The flies were treated with 0.1 mg/mL of MFCC and 0.25 ppm of CP, for 48 hours, and activities of acetylcholinesterase (AChE) (a), superoxide dismutase (SOD) (b), catalase (CAT) (c), and glutathione S-transferase (GST) (d) were determined. The statistical analysis was performed by one-way ANOVA and the Newman-Keuls post hoc test. Significance in relation to group control. The results are expressed as mean ± SEM. ^∗^*p* < 0.05, ^∗∗^*p* < 0.01, ^∗∗∗^*p* < 0.001, and ^∗∗∗∗^*p* < 0.0001 in relation to the control group and ^###^*p* < 0.001 and ^####^*p* < 0.0001 in relation to the CP-treated group.

AChE inhibition is the main mechanism of action for organophosphates. In this study, the organophosphate decreases by 66% (150 to 50 mU/mg of protein) the activity of AChE (Figure 4(a)). This effect was blocked by the presence of MFCC. CP augmented the activity of antioxidant enzymes SOD (Figure 4(b)) and GST (Figure 4(d)). The SOD activity returned to control levels in the presence of fraction. The fraction *per se* increased SOD activity. The association of CP and MFCC did not inhibit the induction in GST activity promoted by CP (Figure 4(d)). Catalase activity was not affected by the treatments (Figure 4(c)).

### 3.8. Evaluation of Isolated Compounds and Chlorpyrifos Effects on Fly Survival

**Figure 5:**
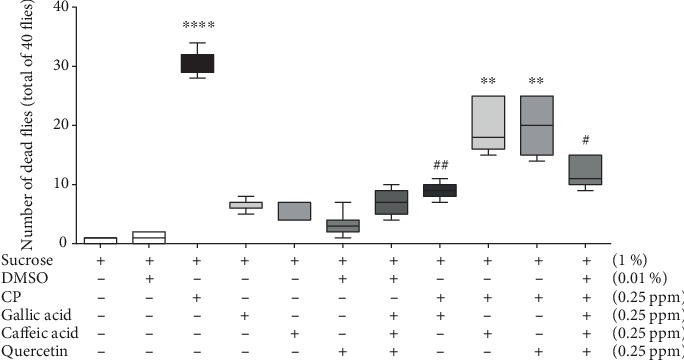
Effects of isolated or combined phenolic compounds in the presence or absence of CP on mortality of *Drosophila melanogaster*. Flies were exposed to gallic acid, caffeic acid, and quercetin in the presence or absence of CP for 48 hours, and mortality was evaluated. The statistical analysis was performed by one-way ANOVA and the Newman-Keuls post hoc test. ^∗∗^*p* < 0.01 and ^∗∗∗∗^*p* < 0.0001 in relation to control; ^#^*p* < 0.05 and ^##^*p* < 0.01 in relation to the CP-treated group.

The methanolic fraction comprises a complex mixture of phytochemical compounds. The effect of majority compounds on CP toxicity was evaluated. As shown in Figure 5, the phenolic compounds gallic acid, caffeic acid, and quercetin did not induce significant fly mortality. In comparison with other compounds, only gallic acid avoided CP toxicity. When all compounds were associated, they protected against CP damage at the same level as gallic acid (Figure 5).

## 4. Discussion

The present study reports the protective effect of the methanolic fraction of leaves of the Brazilian plant *C. campestris* A. St.-Hill on a model of toxicity induced by the organophosphate CP in *Drosophila melanogaster*. CP is an agrochemical compound largely used for controlling pests in crops. In the last decades, the expansion of agriculture has led to the indiscriminate deposition of toxic xenobiotics such as organophosphate in ecosystems leading to human contamination and becoming a global health concern. In animal models and humans, intoxication with organophosphates has been associated with the cholinergic syndrome, neuropathies, and neuropsychiatric disorders and renal injury [39, 40].

In this study, CP exposure resulted in a decrease in fly survival in a concentration-dependent manner. The compound also impaired the locomotor performance of flies. Both effects were not observed when the plant was present. Chlorpyrifos increased reactive oxygen species (ROS) generation and inhibited AChE activity. The fraction blocked those effects thus preventing fly mortality. In the literature, oxidative stress is an important mechanism implied in CP toxicity. Augmented levels of ROS could occur in response to mitochondrial activity inhibition [10]. In other studies, it was demonstrated that CP inhibited complex I and decreased ATP levels in hens [10] and organophosphates caused inhibition of mitochondrial enzymes in rats [41]. Impairment and uncoupling of specific respiratory chain complexes under some stress conditions favor the formation of free radicals. In this regard, our study points out to an impairment of mitochondrial activity as evaluated by a decrease in the fluorescence of resazurin which can be used as an indicator of mitochondrial function [42] once it is converted to a reduced form called resorufin (pink and highly fluorescent) by oxidoreductases in mitochondria.

The augmented levels of MDA in a fly homogenate induced by CP were prevented by the *C. campestris* A. St.-Hill fraction. MDA is a product of lipid peroxidation which occurs in an oxidative stress condition in response to an altered cell redox status. This result together with data from DCF-DA reflects the in vivo antioxidant potential of this fraction. The antioxidant activity is one of the multiple biological effects attributed to phenolic compounds and is related to their metal ion-chelating activity and free radical scavenging activity. The donation of an electron to a free radical from –OH groups attached to the phenolic rings leads to stabilization and inactivation of free radicals [38].

Through HPLC, the presence of phenolic compounds belonging to the phenolic acids and flavonoid groups was identified. To understand the involvement of some of these compounds in the protective action of the methanolic fraction against CP on fly mortality and to observe a possible synergistic effect, quercetin, gallic acid, and caffeic acid alone or in combination were used in concentrations that they are found in fraction composition. Fraction and its compounds did not affect significantly fly survival, and only gallic acid was able to protect the flies against mortality induced by CP. Gallic acid (3,4,5-trihydroxybenzoic acid) comprises a phenolic acid found in different plant families and distributed in almost every part of the plants [43]. The neuroprotective action in different models of neurodegeneration, neurotoxicity, and oxidative stress is described for gallic acid [43, 44].

The inhibition of acetylcholinesterase is the major mechanism implied in CP toxicity. This enzyme hydrolyzes acetylcholine terminating its synaptic action [45]. Although the inhibitory potential of natural phenols on AChE is reported, in this study, the fraction did not affect the activity of this enzyme *per se* but prevented the inhibitory effect of CP on this enzyme. Similarly, natural antioxidants such as kaempferol were able to reestablish AChE activity in brain homogenates [46]. Our data could suggest a possible interaction between CP and some components of the extract, reducing the availability of CP able to bind to the AChE site.

In this work, the organophosphate CP induced the activity of antioxidant enzymes SOD and GST. SOD is an important enzyme with a primary role in eliminating superoxide anions in hydrogen peroxide and oxygen. In insects, seven types of SOD were reported [47]. In *Drosophila*, this enzyme has been associated with resistance to organophosphate [48]. In this study, CP stimulated the activity of SOD. Similarly, the fraction induced the activity of this enzyme. This fact may be in part attributed to the capability of flavonoids to induce the expression of antioxidant enzymes [49] and could contribute to their protective effect. In the presence of the fraction and CP, SOD activity was not altered in relation to control. This result suggests a possible interaction between CP and the fraction, neutralizing their effects on the enzyme.

**Figure 6:**
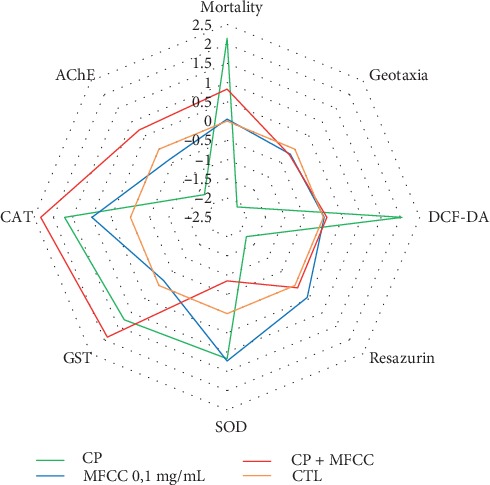
Integrated biomarker response (IBR) index illustrating results obtained in this study. Biomarker results are represented in relation to the control group (CTL). The area above 0 reflects the induction of the biomarker, and that below 0 indicates the reduction of the biomarker.

Like in mammals and in insects, the enzyme glutathione S-transferases (GSTs) are multifunctional enzymes that are responsible for the metabolism and detoxification of both xenobiotic and physiological substances forming water-soluble conjugates facilitating their elimination [50]. Herein, CP alone or in the presence of fraction was able to increase the activity of this enzyme in relation to control. This fact could indicate the involvement of a GST-dependent mechanism in detoxification of CP in flies. An integrated biomarker response (IBR) diagram summarizes the results obtained in this work (Figure 6).

## 5. Conclusion

The present study reports for the first time the protective action of *C. campestris* A. St.-Hill against the toxicity of the organophosphate chlorpyrifos. The plant avoided the inhibition of AChE by CP thus attributing a neuroprotective potential to this plant. Gallic acid was demonstrated to contribute to the protective potential of the fraction in comparison with other phenolic compounds. Therefore, MFCC may be considered a promising source of potential therapeutic agents for the treatment of organophosphate intoxications.

## Figures and Tables

**Table 1:** Composition of the *Croton campestris* A. St.-Hill methanolic fraction.

Compounds	UV (nm)	*Croton campestris*		Equation	*R* ^2^	LOD	LOQ
		mg/g	%			*μ*g/mL	*μ*g/mL
Gallic acid	254	10.57 ± 0.03 a	1.05	*y* = 13629*x* + 1195.8	0.9993	0.024	0.079
Catechin	280	3.89 ± 0.01 b	0.38	*y* = 12407*x* + 1259.6	0.9997	0.007	0.023
Chlorogenic acid	325	19.25 ± 0.01 c	1.92	*y* = 14061*x* + 1325.3	0.9995	0.013	0.042
Caffeic acid	325	27.64 ± 0.2 d	2.76	*y* = 11758*x* + 1359.2	0.9996	0.035	0.115
Rutin	365	6.02 ± 0.03 e	0.60	*y* = 12845*x* + 1065.7	0.9999	0.042	0.138
Quercitrin	365	15.48 ± 0.04 f	1.54	*y* = 13478*x* + 1359.5	0.9994	0.019	0.063
Quercetin	365	30.29 ± 0.01 d	3.02	*y* = 13560*x* + 1192.6	0.9991	0.028	0.092
Kaempferol	365	11.05 ± 0.03 a	1.10	*y* = 14253*x* + 1238.9	0.9997	0.015	0.049

Results are expressed as mean ± standard deviations (SD) of three determinations. Averages followed by different letters differ by the Tukey test at *p* < 0.001.

## Data Availability

The data used to support the findings of this study are available from the corresponding author upon request.

## Supplementary Materials

Supplementary Materials Supplementary material I: effects of exposure to different concentrations of MFCC (0.001, 0.01, 0.1, and 1 mg/mL) on mortality of *Drosophila melanogaster* after 7 days of treatment. The results are represented as mean ± standard error of the mean (SEM). The statistical analysis was performed by one-way ANOVA and the Newman-Keuls post hoc test. Supplementary material II: effects of exposure to different concentrations of CP (0.075, 0.15, 0.25, and 0.30 ppm) in *Drosophila melanogaster* after 48 hours of treatment. The results are represented as mean ± standard error (SEM). The statistical analysis was performed by one-way ANOVA and the Newman-Keuls post hoc test. ^∗∗∗^*p* < 0.001.

## References

[1] Bustos P., Caprettini B., Ponticelli J. (2016). Agricultural productivity and structural transformation. Evidence from Brazil. *American Economic Review*.

[2] Saunders M., Magnanti B. L., Correia Carreira S. (2012). Chlorpyrifos and neurodevelopmental effects: a literature review and expert elicitation on research and policy. *Environmental Health*.

[3] Schopfer L. M., Lockridge O. (2018). Chlorpyrifos oxon promotes tubulin aggregation via isopeptide cross-linking between diethoxyphospho-Lys and Glu or Asp: implications for neurotoxicity. *The Journal of Biological Chemistry*.

[4] Adeyinka A., Kondamudi N. P. (2018). *Cholinergic Crisis*.

[5] Pérez-Aguilar B., Vidal C. J., Palomec G. (2015). Acetylcholinesterase is associated with a decrease in cell proliferation of hepatocellular carcinoma cells. *Biochimica et Biophysica Acta (BBA) - Molecular Basis of Disease*.

[6] Mangas I., Vilanova E., Estévez J. (2016). Neurotoxic effects associated with current uses of organophosphorus compounds. *Journal of the Brazilian Chemical Society*.

[7] Mittler R. (2017). ROS are good. *Trends in Plant Science*.

[8] Lee J. E., Park J. H., Shin I. C., Koh H. C. (2012). Reactive oxygen species regulated mitochondria-mediated apoptosis in PC12 cells exposed to chlorpyrifos. *Toxicology and Applied Pharmacology*.

[9] Lin W.-S., Chen J.-Y., Wang J.-C. (2014). The anti-aging effects of *Ludwigia octovalvis* on *Drosophila melanogaster* and SAMP8 mice. *Age*.

[10] Salama M., El-Morsy D., El-Gamal M., Shabka O., Mohamed W. M. (2014). Mitochondrial complex I inhibition as a possible mechanism of chlorpyrifos induced neurotoxicity. *Annals of Neurosciences*.

[11] Worek F., Thiermann H., Wille T. (2016). Oximes in organophosphate poisoning: 60 years of hope and despair. *Chemico-Biological Interactions*.

[12] Rodrigues N. R., dos Santos Batista J. E., de Souza L. R. (2015). Activation of p38^MAPK^ and NRF2 signaling pathways in the toxicity induced by chlorpyrifos in *Drosophila melanogaster*: protective effects of *Psidium guajava pomífera* L. (Myrtaceae) hydroalcoholic extract. *Arabian Journal of Chemistry*.

[13] de Andrade Franco J. L. (2013). O conceito de biodiversidade e a história da biologia da conservação: da preservação da wilderness à conservação da biodiversidade. *História (São Paulo)*.

[14] Coutinho H. D. M., Matias E. F. F., Santos K. K. A. (2011). Modulación de la resistencia a norfloxacina de Staphylococcus aureus por Croton A. campestris y Ocimum gratissimum L.. *Biomédica*.

[15] de Almeida T. S., Rocha J. B. T., Rodrigues F. F. G., Campos A. R., da Costa J. G. M. (2013). Chemical composition, antibacterial and antibiotic modulatory effect of *Croton campestris* essential oils. *Industrial Crops and Products*.

[16] Júnior F. E. B., de Oliveira D. R., Bento E. B. (2013). Antiulcerogenic activity of the hydroalcoholic extract of leaves of Croton campestris A. St.-Hill in rodents. *Evidence-Based Complementary and Alternative Medicine*.

[17] Matias E. F. F., Santos K. K. A., Almeida T. S., Costa J. G. M., Coutinho H. D. M. (2011). Phytochemical prospection and modulation of aminoglycoside antibiotic activity by *Croton campestris* A.. *Chemotherapy*.

[18] El Babili F., Fabre N., Moulis C., Fouraste I. (2006). Molluscicidal activity against Bulinus truncatus of Croton campestris. *Fitoterapia*.

[19] de S Falcão H., Lima I. O., dos Santos V. L. (2005). Review of the plants with anti-inflammatory activity studied in Brazil. *Revista Brasileira de Farmacognosia*.

[20] Júnior F. E. B., Macedo G. E., Zemolin A. P. (2016). Oxidant effects and toxicity of *Croton campestris* in *Drosophila melanogaster*. *Pharmaceutical Biology*.

[21] Tian Y., Liimatainen J., Alanne A.-L. (2017). Phenolic compounds extracted by acidic aqueous ethanol from berries and leaves of different berry plants. *Food Chemistry*.

[22] Kalender Y., Kaya S., Durak D., Uzun F. G., Demir F. (2012). Protective effects of catechin and quercetin on antioxidant status, lipid peroxidation and testis-histoarchitecture induced by chlorpyrifos in male rats. *Environmental Toxicology and Pharmacology*.

[23] Kaur S., Singla N., Dhawan D. K. (2019). Neuro-protective potential of quercetin during chlorpyrifos induced neurotoxicity in rats. *Drug and Chemical Toxicology*.

[24] Matos F. J. A. (1997). *Introdução à fitoquímica Experimental*.

[25] Boligon A. A., De Brum T. F., Frolhich J. K., Froeder A. L. F., Athayde M. L. (2012). HPLC/DAD profile and determination of total phenolics, flavonoids, tannins and alkaloids contents of Scutia buxifolia Reissek stem bark. *Research Journal of Phytochemistry*.

[26] Boligon A. A., Janovik V., Boligon A. A. (2013). HPLC analysis of polyphenolic compounds and antioxidant activity *inNasturtium officinale*. *International Journal of Food Properties*.

[27] Paula M. T., Zemolin A. P., Vargas A. P. (2014). Effects of Hg (II) exposure on MAPK phosphorylation and antioxidant system in *D. melanogaster*. *Environmental Toxicology*.

[28] Bland N. D., Robinson P., Thomas J. E., Shirras A. D., Turner A. J., Isaac R. E. (2009). Locomotor and geotactic behavior of Drosophila melanogaster over-expressing neprilysin 2. *Peptides*.

[29] Ellman G. L., Courtney K. D., Andres V., Featherstone R. M. (1961). A new and rapid colorimetric determination of acetylcholinesterase activity. *Biochemical Pharmacology*.

[30] Kostyuk V. A., Potapovich A. I. (1989). Superoxide--driven oxidation of quercetin and a simple sensitive assay for determination of superoxide dismutase. *Biochemistry International*.

[31] Aebi H. (1984). Catalase in vitro. *Methods in Enzymology*.

[32] Habig W. H., Jakoby W. B. (1981). [51] Assays for differentiation of glutathione S-transferases. *Methods in Enzymology*.

[33] Bradford M. M. (1976). A rapid and sensitive method for the quantitation of microgram quantities of protein utilizing the principle of protein-dye binding. *Analytical Biochemistry*.

[34] Macedo G. E., Gomes K. K., Rodrigues N. R. (2017). *Senecio brasiliensis* impairs eclosion rate and induces apoptotic cell death in larvae of *Drosophila melanogaster*. *Comparative Biochemistry and Physiology Part C: Toxicology & Pharmacology*.

[35] Saraiva M. A., da Rosa Ávila E., da Silva G. F. (2018). Exposure of *Drosophila melanogaster* to Mancozeb induces oxidative damage and modulates Nrf2 and HSP70/83. *Oxidative Medicine and Cellular Longevity*.

[36] Karatas F., Karatepe M., Baysar A. (2002). Determination of free malondialdehyde in human serum by high-performance liquid chromatography. *The New England Journal of Medicine*.

[37] Beliaeff B., Burgeot T. (2002). Integrated biomarker response: a useful tool for ecological risk assessment. *Environmental Toxicology and Chemistry*.

[38] Nimse S. B., Pal D. (2015). Free radicals, natural antioxidants, and their reaction mechanisms. *RSC Advances*.

[39] Jokanović M. (2018). Neurotoxic effects of organophosphorus pesticides and possible association with neurodegenerative diseases in man: a review. *Toxicology*.

[40] Prudente I. R. G., Cruz C. L., de Carvalho Nascimento L., Kaiser C. C., Guimarães A. G. (2018). Evidence of risks of renal function reduction due to occupational exposure to agrochemicals: a systematic review. *Environmental Toxicology and Pharmacology*.

[41] Masoud A., Kiran R., Sandhir R. (2009). Impaired mitochondrial functions in organophosphate induced delayed neuropathy in rats. *Cellular and Molecular Neurobiology*.

[42] Zhang H., Du G., Zhang J. (2004). Assay of mitochondrial functions by resazurin in vitro. *Acta Pharmacologica Sinica*.

[43] Daglia M., Di Lorenzo A., Nabavi S. F., Talas Z. S., Nabavi S. M. (2014). Polyphenols: well beyond the antioxidant capacity: gallic acid and related compounds as neuroprotective agents: you are what you eat!. *Current Pharmaceutical Biotechnology*.

[44] Mansouri M. T., Farbood Y., Sameri M. J., Sarkaki A., Naghizadeh B., Rafeirad M. (2013). Neuroprotective effects of oral gallic acid against oxidative stress induced by 6-hydroxydopamine in rats. *Food Chemistry*.

[45] Thapa S., Lv M., Xu H. (2017). Acetylcholinesterase: a primary target for drugs and insecticides. *Mini-Reviews in Medicinal Chemistry*.

[46] Hussein R., Mohamed W., Omar H. A. (2018). A neuroprotective role of kaempferol against chlorpyrifos-induced oxidative stress and memory deficits in rats via GSK3*β*-Nrf2 signaling pathway. *Pesticide Biochemistry and Physiology*.

[47] Kobayashi Y., Nojima Y., Sakamoto T. (2019). Comparative analysis of seven types of superoxide dismutases for their ability to respond to oxidative stress in Bombyx mori. *Scientific Reports*.

[48] Ling S., Zhang H. (2013). Influences of chlorpyrifos on antioxidant enzyme activities of Nilaparvata lugens. *Ecotoxicology and Environmental Safety*.

[49] Wang L., Li Y. M., Lei L. (2015). Cranberry anthocyanin extract prolongs lifespan of fruit flies. *Experimental Gerontology*.

[50] Hernandez E. P., Kusakisako K., Talactac M. R. (2018). Glutathione S-transferases play a role in the detoxification of flumethrin and chlorpyrifos in Haemaphysalis longicornis. *Parasites and Vectors*.

