# Supplementary material for: Croton campestris A. St.-Hill Methanolic Fraction in a Chlorpyrifos-Induced Toxicity Model in Drosophila melanogaster: Protective Role of Gallic Acid
Source: Oxid Med Cell Longev. 2020 Mar 22;2020:3960170. doi: 10.1155/2020/3960170 (PMC7121785; doi:10.1155/2020/3960170)

**Supplementary material I.** Effects of exposure to different concentrations of MFCC (0.001, 0.01, 0.1, 1 mg/mL) in mortality of *Drosophila melanogaster* after 7 days of treatment. The results are represented as mean ± standard error of the (SEM). The statistic was performed by One-Way ANOVA and post hoc Newman-keuls test.
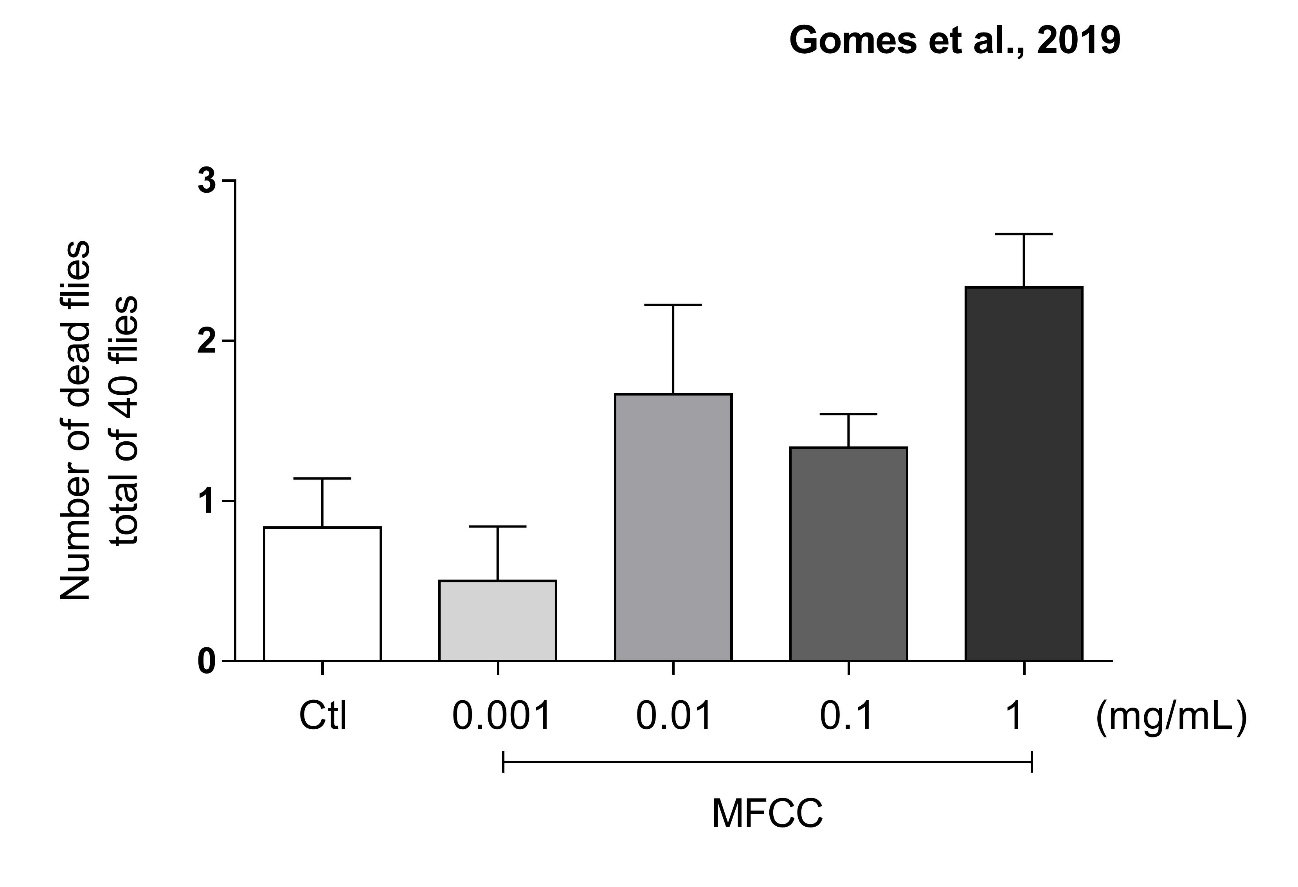


**Supplementary material II.** Effects of exposure to different concentrations of CP (0.075, 0.15, 0.25, 0.30 ppm) in *Drosophila melanogaster* after 48 hours of treatment. The results are represented as mean ± standard error (SEM). The statistic was performed by One-Way ANOVA and post hoc Newman-keuls test. ***p < 0.001.


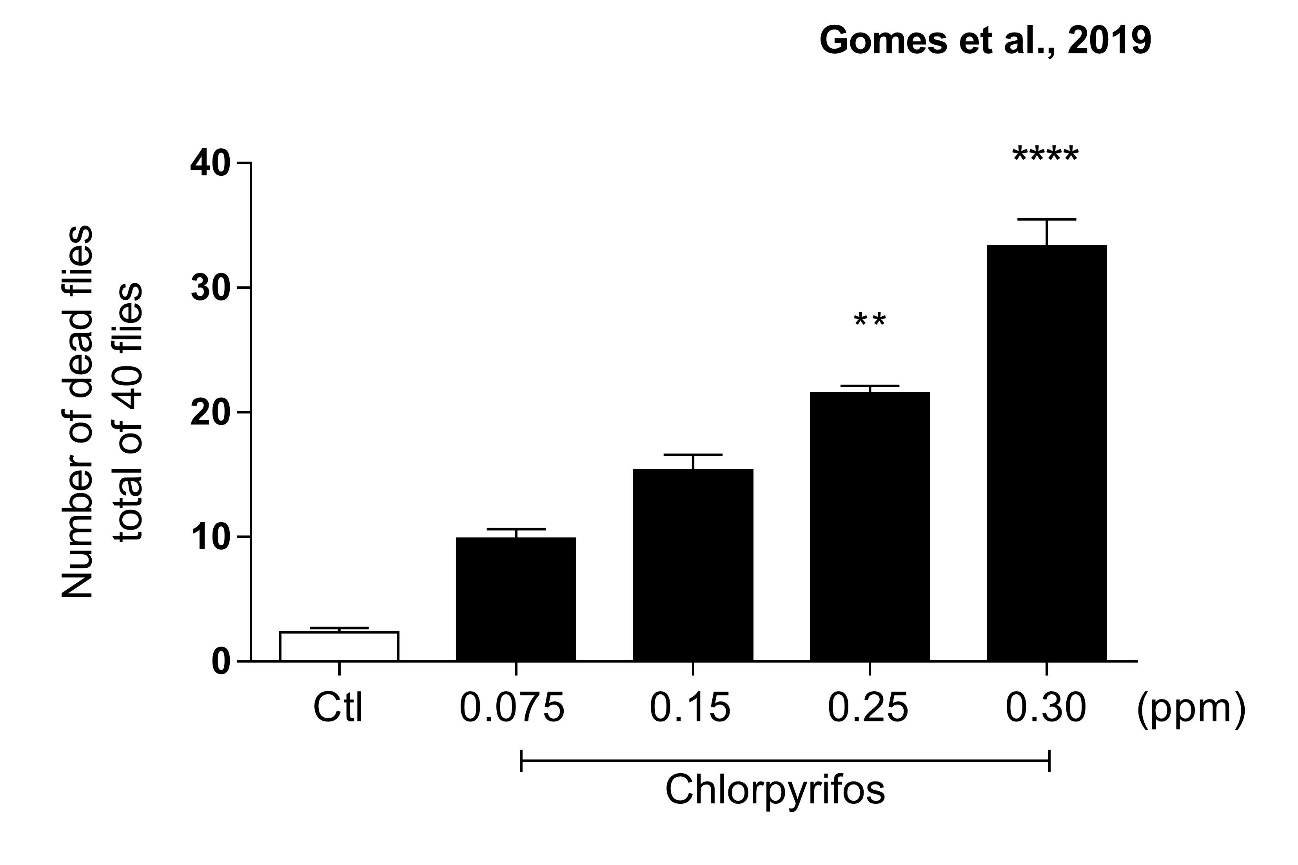

Supplement: Supplementary Materials — Supplementary material I: effects of exposure to different concentrations of MFCC (0.001, 0.01, 0.1, and 1 mg/mL) on mortality of Drosophila melanogaster after 7 days of treatment. The results are represented as mean ± standard error of the mean (SEM). The statistical analysis was performed by one-way ANOVA and the Newman-Keuls post hoc test. Supplementary material II: effects of exposure to different concentrations of CP (0.075, 0.15, 0.25, and 0.30 ppm) in Drosophila melanogaster after 48 hours of treatment. The results are represented as mean ± standard error (SEM). The statistical analysis was performed by one-way ANOVA and the Newman-Keuls post hoc test. ∗∗∗p < 0.001. [file 3960170.f1.docx]
